# Interface-Resolved Network of Protein-Protein Interactions

**DOI:** 10.1371/journal.pcbi.1003065

**Published:** 2013-05-16

**Authors:** Margaret E. Johnson, Gerhard Hummer

**Affiliations:** Laboratory of Chemical Physics, National Institute of Diabetes and Digestive and Kidney Diseases, National Institutes of Health, Bethesda, Maryland, United States of America; University of Heidelberg, Germany

## Abstract

We define an interface-interaction network (IIN) to capture the specificity and competition between protein-protein interactions (PPI). This new type of network represents interactions between individual interfaces used in functional protein binding and thereby contains the detail necessary to describe the competition and cooperation between any pair of binding partners. Here we establish a general framework for the construction of IINs that merges computational structure-based interface assignment with careful curation of available literature. To complement limited structural data, the inclusion of biochemical data is critical for achieving the accuracy and completeness necessary to analyze the specificity and competition between the protein interactions. Firstly, this procedure provides a means to clarify the information content of existing data on purported protein interactions and to remove indirect and spurious interactions. Secondly, the IIN we have constructed here for proteins involved in clathrin-mediated endocytosis (CME) exhibits distinctive topological properties. In contrast to PPI networks with their global and relatively dense connectivity, the fragmentation of the IIN into distinctive network modules suggests that different functional pressures act on the evolution of its topology. Large modules in the IIN are formed by interfaces sharing specificity for certain domain types, such as SH3 domains distributed across different proteins. The shared and distinct specificity of an interface is necessary for effective negative and positive design of highly selective binding targets. Lastly, the organization of detailed structural data in a network format allows one to identify pathways of specific binding interactions and thereby predict effects of mutations at specific surfaces on a protein and of specific binding inhibitors, as we explore in several examples. Overall, the endocytosis IIN is remarkably complex and rich in features masked in the coarser PPI, and collects relevant detail of protein association in a readily interpretable format.

## Author Summary

Much of the work inside the cell is carried out by proteins interacting with other proteins. Each edge in a protein-protein interaction network reflects these functional interactions and each node a separate protein, creating a complex structure that nevertheless follows well-established global and local patterns related to robust protein function. However, this network is not detailed enough to assess whether a particular protein can bind multiple interaction partners simultaneously through distinct interfaces, or whether the partners targeting a specific interface share similar structural or chemical properties. By breaking each protein node into its constituent interface nodes, we generate and assess such a detailed new network. To sample protein binding interactions broadly and accurately beyond those seen in crystal structures, our method combines computational interface assignment with data from biochemical studies. Using this approach we are able to assign interfaces to the majority of known interactions between proteins involved in the clathrin-mediated endocytosis pathway in yeast. Analysis of this interface-interaction network provides novel insights into the functional specificity of protein interactions, and highlights elements of cooperativity and competition among the proteins. By identifying diverse multi-protein complexes, interface-interaction networks also provide a map for targeted drug development.

## Introduction

Protein-protein interaction (PPI) networks aim to capture the interactions between proteins that mediate many of their molecular functions [1]–[3]. However, with one node per protein and one edge per binary interaction, PPIs provide only a coarse rendering of the nuanced molecular level interactions. With exposed surfaces ranging from tens to hundreds of residues, proteins may present multiple distinct binding interfaces. Each interface can mediate binding to a single partner, or to multiple partners. The cooperative or competitive character of these interactions tunes protein availability in the cell, the formation of higher order complexes, and ultimately many important biological functions. Proteins with multiple binding interfaces can bring together distinct partners to assemble transient or permanent complexes. In contrast, multiple distinct partners competing for a single shared interface may function to connect disparate functional modules in the cell [4], [5], with such competitive binding having arisen, for instance, as a result of gene duplication [6], [7]. Distinguishing the types of binding interfaces a protein uses for each interaction partner is a key step to resolving the cooperativity inherent in functional protein interactions. Moreover, a protein interaction network with resolved interfaces helps to connect gene mutations with disease [8], and to identify possible drug targets, with inhibitors of protein-protein binding receiving increasing attention [9]–[12]. In particular, by targeting interfaces shared in multiple binding interactions, one may be able to shut down entire pathways, whereas targeting more isolated interactions offers a route for a more measured intervention. Assigning interfaces to protein interactions thus has both fundamental and practical relevance.

**Figure 1:**
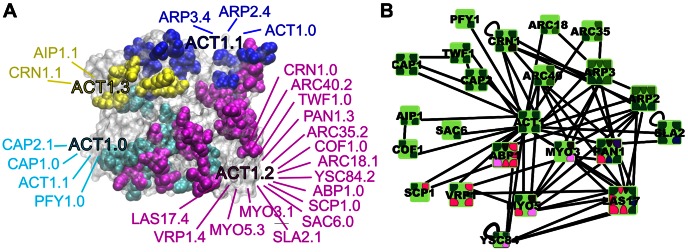
Distinct binding interfaces and IIN of yeast actin protein ACT1, and its corresponding PPI network with interfaces overlaid. (a) Actin surface structure (grey; PDB structure 1YAG) with binding interfaces in van-der-Waals representation and binding partner interfaces listed in matching colors, indicating the IIN for the actin protein. Residues in interfaces ACT1.0 (cyan), ACT1.1 (blue) and ACT1.2 (magenta) were determined from crystal structures of complexes (PDB structures 3J0S and 3LUE for ACT1.0 and ACT1.1, and structures 2A3Z, 2A41, 3LUE and 3J0S for ACT1.2). In the absence of structures for the ACT1.3 (yellow) complex, we used the results of genetic studies [61] to highlight surface residues of actin subunit IV that are both essential for binding to AIP1 and not involved in COF1 binding mediated by the ACT1.2 interface. (b) Sub-network of protein interactions involving actin-binding proteins of CME with interfaces defined. Colors indicate specific domain types listed in Figure 3.

To refine the coarse protein-protein interaction network and to capture these important structural and chemical aspects of interactions [5] requires the identification of the binding domains or interfaces on each protein. Importantly, one needs to distinguish on the basis of clear rules between binding partners that target overlapping or distinct surface regions. By systematically cataloguing these details it is possible to create not only a map of shared versus distinct binding interactions [5], [13], [14], but an entirely new sub-network of the protein interaction network, as we do here. A PPI network with interfaces overlaid on the proteins highlights the number of interfaces each protein uses to mediate binding and the number of binding partners per interface (see Figure 1b). An interface-interaction network (IIN) is what one gets by visualizing the protein interface connectivity as separated from the underlying PPI network. Unlike in the PPI network representation, in the IIN representation distinct patterns of connectivity between interfaces emerge, and this network topology can be analyzed to yield insight into the specificity and possible cooperation and competition of protein interactions.

Although the importance of structural details in protein interactions has led to increasing efforts to identify protein-binding interfaces in a systematic way [13], [15]–[18], PPI networks with interfaces overlaid on them and detailed IINs have not previously been created. Earlier studies have used protein structures combined with homology modeling [5], genomic data [19], and docking algorithms [20]–[22], to both assign and infer [23] binding interfaces. While it would be possible to construct an IIN from the residue level details collected from some structural data [21], [23], both the accuracy and coverage of the network would be limited by the errors inherent to homology modeling or docking methods, and by the fact that crystallized protein complexes cover only a small percentage of known protein-protein interactions [20]. In particular, using structural homology to infer binding partners provides important guidance but may overestimate the number of binding partners because structural homologs reflect evolution but not necessarily shared functions [24], and small differences in sequences can separate specific from non-specific binding [25]. Proteins with disordered regions and without structural or domain information would be absent from the network, thus sampling only subsets of interaction types within the proteome. The presence of false positives in the IIN would obscure the diverse patterns that emerge in the network and distinguish the network structure from that of the parent PPI. The topology of a network reflects functional pressures acting to connect nodes in specific ways, whether the nodes are proteins, interfaces, or airports. One force shaping the PPI network is the need to transmit information across diverse functional modules, resulting in a giant connected component with few unconnected proteins. Differences in the IIN topology imply different functional and physical forces acting on the component interfaces.

In order to analyze the specificity, cooperativity, and topological properties of an IIN one requires both an accurate assessment of shared and distinct binding interfaces and a dense collection of protein-protein interactions. Therefore, we here combine both structure-based computational approaches with literature-curated biochemical data to build an IIN for the proteins involved in clathrin-mediated endocytosis (CME) in yeast [26]. CME is a central pathway for internalizing cargo such as nutrients and signaling molecules into the cell. Assigning the interfaces mediating each protein interaction would be severely limited if we relied on structural data alone, as several of these interactions are mediated by short peptide motifs on disordered regions. Furthermore, any uncertainty associated with using models of possible domain interactions is completely bypassed by exploiting the wealth of established domain and residue information known from biochemical experiments. Through this process, we are able to carefully evaluate whether a protein's interaction partners bind to the same interface, or to distinct interfaces. Figure 1a illustrates the results of the interface assignment for the yeast actin protein, ACT1, and Figure 1b shows the PPI network with interfaces overlaid for the subset of actin binding proteins. This representation captures both the ability of actin to bind several proteins simultaneously through four distinct interfaces, and the competition between multiple proteins to bind each of these interfaces. The resolution of this network exceeds in two important ways that of networks obtained by only defining PPI network edges as competing. First, because all of actin's binding partners compete with at least a few others to bind actin, all PPI edges from actin would be marked as competing. Therefore it would not be possible to distinguish which of actin's binding partners can bind at the same time. Second, because an edge connects two proteins, a network with edges marked as competing does not clarify which protein surface (say, actin's or its partner's) is actually shared, as sharing can occur on one or both proteins.

In addition to collecting detailed data on protein structures, a particular advantage of our curated approach is to eliminate false positives from the PPI by creating a coherent and consistent picture of the protein interactions. We identify the specific mechanism mediating an observed protein-protein interaction and determine whether the interaction is direct or indirect. Of particular concern are indirect interactions, mediated through intervening proteins, because they are not always distinguishable from direct interactions in high-throughput affinity purification/mass spectrometry (AP/MS) [3], protein-fragment complementation assay (PCA) [27] or, to a lesser extent, yeast two hybrid (Y2H) experiments [3]. Literature sources also document protein pairs tested and found to not bind. Therefore, by curating the literature we do not predict new interactions but we do remove spurious interactions. We also compile the number and types of experiments used to identify the interfaces in each protein-protein interaction, as the interfaces can vary from high-resolution selections of specific residues to low-resolution large regions of the protein. This compilation provides a starting point for improving the resolution of the structural interaction.

The CME network constructed and characterized in this way reveals significant complexity with permanent and dynamic assemblies of few or many proteins, a mixture of binding modes with both shared and distinct binding, and both large and small binding interfaces. This detailed information is necessary for building models of protein-protein interactions where both competitive and cooperative binding reactions contribute to function. The accuracy and coverage of the protein IIN we have generated allows us to draw generalizable insights about the structure of the IIN, the overlap of binding interfaces, the identification of indirect interactions, and the implications towards the biological functions with the parent PPI. Compiling this information for more parts of PPI networks will help prune indirect and spurious interactions, highlight areas of poorly resolved structural and biochemical characterization, and facilitate investigation of the physical and evolutionary origins of the IIN topology and in turn of protein binding.

The aims of the present work are (1) to develop a general framework for the construction of IINs from a combination of structural and biochemical data that measure the support of proposed protein interactions; (2) to characterize the general network properties of the resulting endocytosis IIN as compared to PPI networks and randomized networks, and (3) to demonstrate the applications of the IIN, including as a resource for predicting response to mutation and to specific binding inhibitors. At the network level, we examine whether the IIN retains the complex characteristics of the PPI network, including a high connectivity, hub structures, local clustering, and a scale-free character manifested in power-law distributions of the number of binding partners. We also quantify the fragmentation of the fully connected parent PPI network into separate interface modules at the IIN level. We provide several examples for the use of the IIN in selecting possible drug targets and in predicting the effects of mutations by identifying specific pathways of communication between proteins via their interfaces. For the CME proteins we discuss the central role of SH3 domains and multi-interface proteins. We emphasize that the process of assigning protein interfaces has generated not only a useful map of interactions among these highly-studied proteins but has highlighted the difficulties associated with trying to make automated assignments, including overlapping residues and inconsistencies between sources. Therefore, we discuss the insights derived from our interface procurement process that are relevant for high-throughput methods of interface determination.

## Results

### Construction of curated PPI network

As a first step, we constructed a curated PPI network of 56 proteins involved in CME in yeast [26]. Following the approach described in Methods, we first combined 337 edges downloaded from BioGRID [28] via the Saccharomyces Genome Database (SGD) [29] and 49 additional distinct edges collected from IntAct, MINT, DIP, and BIND. 177 edges had interfaces assigned to both proteins in the interaction and nine additional edges were added from literature evidence. We note that for these 56 proteins, we observed significant overlap in the interactions reported in each protein-protein interaction database, as listed in Table 1. Of the assigned protein-protein interactions, sixteen had two binding modes, and two had three binding modes, resulting in a total of 206 assigned interface-interface interactions from 186 assigned protein-protein interactions. We removed 35 edges from the original network because they were suspected to be indirect, shown not to bind in further experiments, or they occurred only in a study of yeast prions, suggesting that the observed binding may not normally be functional. For 28 interactions identified in multiple high-throughput studies no evidence from the literature was found to assign interfaces, and 145 interactions were found only in one reference without sufficient information to assign an interface. Nearly all of the 145 unassigned interactions that were implicated in a single experiment came from high-throughput studies, and because the proteins in the CME subset form the connected clathrin coat and actin patch together, many of these observed interactions could be indirect.

**Table 1:** Protein-protein interactions by database.

Database	Number of interactions	% of interactions in BioGRID	% of BioGRID in this database	Number of interactions not in our original database
**IntAct**	213	89%	63%	21
**MINT**	230	87%	68%	22
**DIP**	205	90%	61%	14
**BIND**	84	75%	25%	26

BioGRID had 337 interactions between the set of 56 proteins. Of the 5 databases, BioGRID contained the most edges, with high coverage of interactions in the other 4 databases. The interactions missing from BioGRID did not arise due to missed references (except for 3 studies of functional rather than physical associations) but due to missed interactions in the same references. The other 4 databases contained a total of 69 interactions not present in BioGRID, and 52 not present in our original database that had been augmented by added edges and through curation of the SH3/PRD and kinase references. Of these 52 interactions, three were removed for erroneous citations, 20 were found only through functional association, and therefore removed, and the remainder were observed in only a single probe of physical interactions.

**Figure 2:**
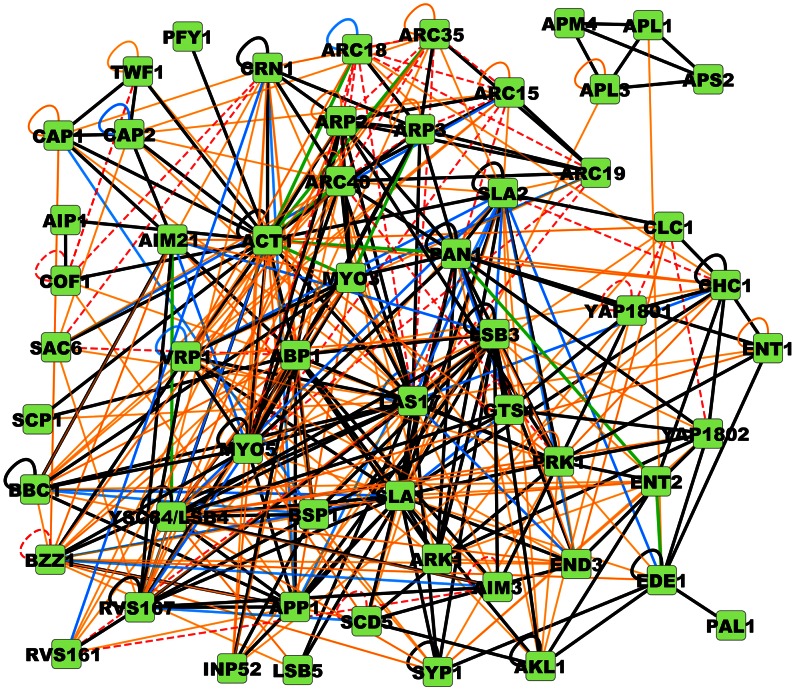
PPI network with 56 clathrin associated proteins and the 386 interactions downloaded from BioGRID, IntAct, MINT, DIP, and BIND. Also shown are the 9 added interactions not found in the PPI databases. Black and green edges had interfaces assigned, with green edges added to the network using literature data. Blue edges were interactions identified in more than one experiment but lacked sufficient information to assign interfaces. Orange edges are interactions cited from a single reference and had insufficient evidence to assign an interface. Red edges were suspected to be indirect or shown in other experiments not to bind. All network figures generated by Cytoscape [62].

**Figure 3:**
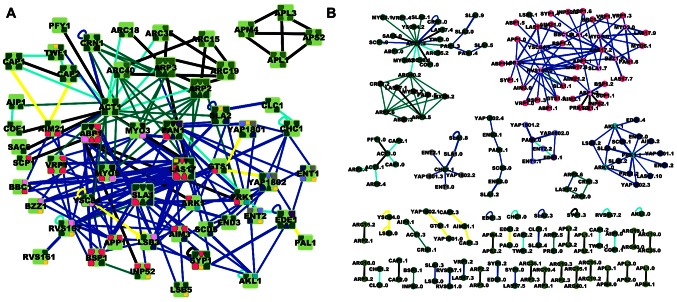
PPI network for endocytosis with interfaces assigned and corresponding IIN. **a)** Each protein (light green square) is shown with all of its distinct interfaces colored according to domain type. Dark and light pink indicate PRD and SH3 domains, respectively. Dark and light purple indicate EH domains and NPF motifs. Dark and light blue indicate phosphorylation sites and kinase domains. Clathrin boxes are shown in gray, and acidic domains in brown. Light green indicates subunit-subunit interfaces defined from a multi-subunit complex, and the rest are colored dark green. Yellow squares indicate membrane binding domains. The edges are colored according to the experimental evidence supporting the interface assignments. Black edges are crystal structures, blue edges had both interface domains resolved, generally via protein truncation or mutation, and thicker blue lines were shown to bind in vivo. Dark green lines had one interface resolved and the other inferred, and for cyan both were inferred. Yellow lines indicate interactions speculated to be distinct interfaces. Details and references are in Table S1. **b)** The resulting IIN with interfaces colored as in 3a.

The differing support for the CME protein interactions is represented visually in Figure 2 and collected along with specific details of their assignment in tabulated form in Table S1. The tabulated list contains all the currently known interactions between these 56 CME proteins and the interface assignment status effectively ranks them in terms of their reliability. Interactions with interfaces assigned are further classified in Figure 3 according to the experimental data used to make the assignment. The blue edges in the Figure 2 network are unresolved interactions that have the most evidence (more than one study) supporting their potential functionality in the cell. The red edges are most likely to be artifacts of the experimental probes of their interaction, on the basis of evidence listed in Table S1. Of the red ‘false positive’ edges, most were indirectly interacting through a larger complex. A few had been shown in detailed biochemical characterizations not to bind to one another.

In Figure 3 we also distinguish the amount and type of evidence used to support each interface assignment by coloring each edge. The number of interface interactions assigned directly from crystal structures is shown in black, and represents a minor fraction of the total assignments for this network. For blue edges, both interfaces were resolved using biochemical studies, typically by truncating or mutating the constituent proteins. We note that although these binding interactions have been tested in vitro and in some cases in vivo (thicker blue lines), some of the interfaces encompass large folded domains rather than specific surface binding residues. These domains could therefore be segregated further into more than one interface given additional resolution of the specific residues involved in each interaction. Green and cyan edges had one or both interface inferred. For these assignments we relied on homology to other proteins either through sequence, function or crystal structure. Alternatively, a lack of competition for binding to a surface or a lack of any structural or functional homology was sometimes used to infer distinct vs shared interfaces. Finally, the yellow edges are speculated to be distinct interfaces due to a lack of observed similarity to known partners or domains, and as such have the weakest support.

**Figure 4:**
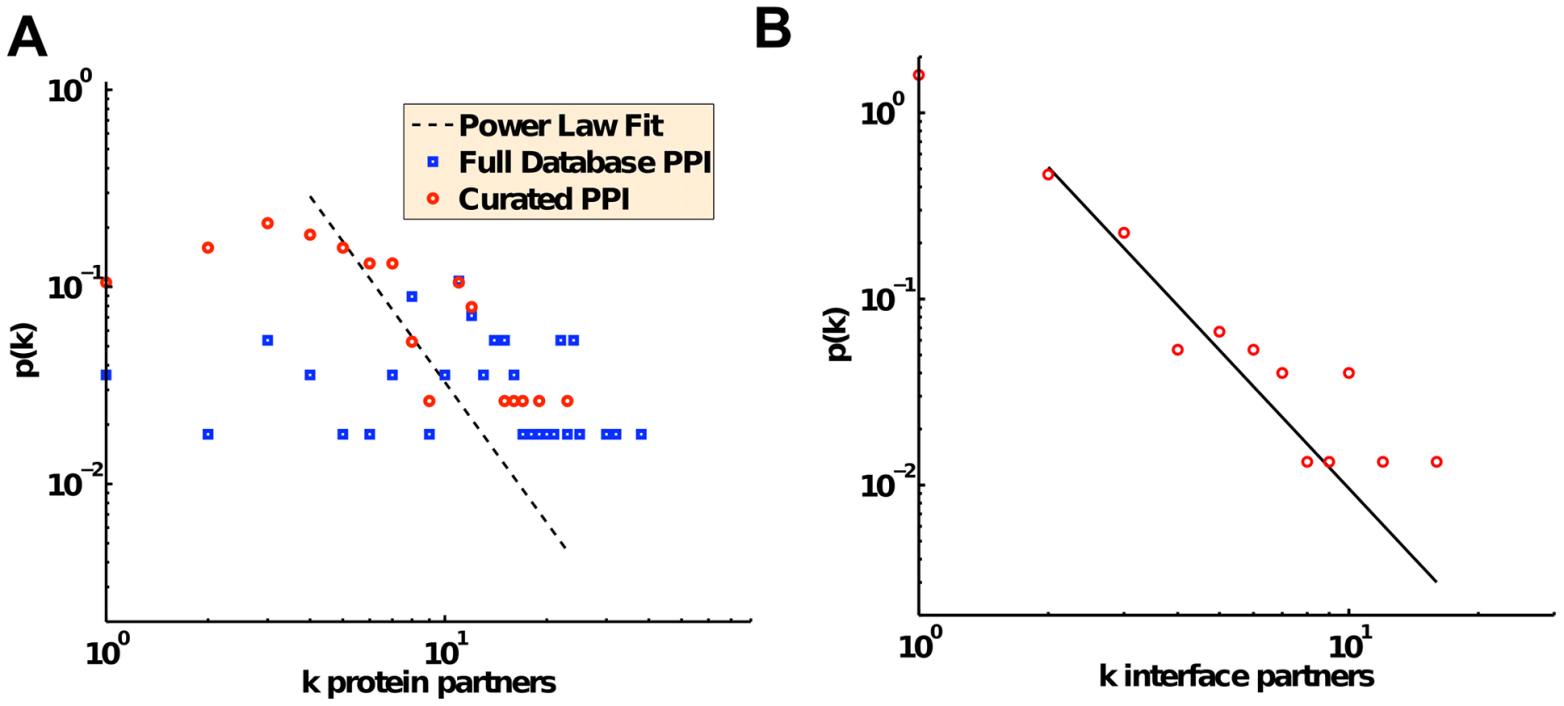
Degree distribution for the endocytosis network. **a)** Endocytosis PPI network. Blue data are for the full interaction network downloaded from the BioGrid, IntAct, MINT, DIP, and BIND databases, before curating the interfaces. The red data are for the protein-protein interaction network with interfaces assigned (Figure 2b). For the curated PPI, a power-law distribution was best fit with k_min_ = 4 and γ = 2.37 (black dashed line), but the resulting p-value is less than 0.05, implying that the hypothesis of a power law density for the data is not accurate. The full database PPI can only be fit with k_min_ = 11, leaving only about half the data points. **b)**. Endocytosis IIN. A power law (solid line) best fits this data with a k_min_ = 2 and γ = 2.47, giving a p-value of 0.26 that implies good consistency with a power-law density.

We determined the degree distributions of both the original and the curated PPI networks, as a statistical measure of the number of interaction partners of each node. Upon going from the full combined-database PPI network (plus the added 9 edges; see Methods) to the curated PPI network, the decrease in total edges results in a less dense network in which the average number of partners per protein dropped significantly from 13.5 to 6.4. Although large PPI networks typically have degree distributions characterized as power law or truncated power laws [30], [31], neither the curated PPI nor the combined-database PPI have degree distributions statistically consistent with a power law density (Figure 4a). The deviations from a power law could be due to the small size of the two networks (only 56 proteins) and the fact that they are all part of a functionally related module. Such deviations would be exacerbated in the original un-curated network, where the distribution is more uniform for N<10, by spurious and likely indirect interactions within the set of proteins absent in the curated PPI network. Lastly, both of the original and curated endocytosis PPI networks had high clustering coefficients (0.56 and 0.46, respectively; see Methods) indicating that proteins that interacted had partners that were likely to interact with one another. Not surprisingly, the clustering coefficient of 0.28 in a full yeast PPI collected from several large-scale studies in yeast [3], [27] is lower, since the CME proteins were specifically chosen to be part of the same functional module.

### Protein interface properties

The majority of proteins in this endocytosis network have multiple interfaces, with an average number of 3.5 distinct binding interfaces per protein. The correlation between protein size and the number of interfaces is quite weak (R = 0.26). One reason for this weak correlation is that several of these proteins have additional binding partners outside the CME network module considered here. Another reason is the size of the interfaces varies broadly, from just a few residues (for NPF motifs [32]) to hundreds of residues (e.g., the clathrin-clathrin leg binding [33]). For example, LAS17 is a medium sized protein at 633 residues that has the most interfaces thanks in part to five short proline-rich domains (PRDs) we assigned as distinct interfaces due to their specificity towards different binding partners [34], [35]. The black edges in Figure 3 connect interfaces determined through crystal structures that therefore are defined by a subset of non-contiguous residues. Interfaces from biochemical studies tend to lack single residue resolution but instead span stretches of residues or complete domains. In the 3D protein structure, only a fragment of these residues would be expected to contact binding partners and as such some of these interfaces could be split or refined further.

### Comparison of PPI network and IIN

It is interesting to compare the change in network properties between the curated PPI network (shown in Figure 3a with interfaces overlaid) and its IIN (Figure 3b). In general, a PPI network and its IIN should have equal numbers of edges, but it is possible for an IIN to have more edges if a pair of interacting proteins has multiple modes of binding to one another. Proteins that act as alternating subunits in a symmetric complex, for example, will contact two copies of the same partner through distinct interfaces. The CME IIN contains several instances of multiple binding modes, resulting in an increase in edges from the PPI. Such distinct modes for the same two proteins to bind one another can act as a regulatory mechanism controlling the accessibility of surfaces on the protein, or as sources of extra stability to the protein-protein interaction. For example, the protein CRN1 contains two distinct actin-binding domains that bind separate regions on the actin surface and are modulated by the nucleotide bound state of actin. Through these multiple binding modes, CRN1 can have opposite roles in either inhibiting or activating the severing of actin filaments [36]. In another example, the SH3 domain of LSB3 binds three distinct PRDS on LAS17. The PRDS on LAS17 follow one after another and the flexibility to bind any one of them to the SH3 domain of LSB3 could help stabilize the binding interaction at different geometries as part of the higher-order actin patch assembly.

The IIN contains more nodes than the PPI, with each node now representing a distinct interface rather than a protein. In general, one would expect such an increase because proteins are known to have evolved multiple domains or interfaces to bind specific partners. The increase in nodes is much greater than the increase in edges from the PPI to the IIN, and therefore the IIN is substantially less densely connected than the PPI, with the average degree dropping from 6.4 to 2.06. In this now sparsely connected network, the clustering coefficient has dropped from 0.46 in the PPI to zero in the IIN. To quantify the significance of this result we generated randomized versions of the IIN that maintain the same number of nodes and the same degree distribution. We find that the randomized networks have distinctly higher clustering coefficients than the IIN (Table 2), suggesting that the structure of the IIN has evolved against having interfaces that bind to one another sharing the same partners. This is in contrast to the PPI network, where the relatively large clustering coefficient reflects the likelihood that two proteins that interact with one another share interaction partners.

**Table 2:** Local IIN structural properties.

	Randomized	Observed
**C_local_**	0.01±0.006	0 (p = 0.008)
**C_global_**	0.025±0.01	0 (p = 0.008)
**4-node hubs**	0.43±0.02	0.56 (p<0.0008)
**4-node chains**	0.54±0.02	0.37 (p<0.0008)
**4-node squares**	0.0028±0.001	0.061 (p<0.0008)

The clustering coefficients and the percentages of 4-node motifs present in the observed CME IIN are compared with 1200 randomized versions of the network that preserve the same degree distribution. The values in parentheses are the p-values for the hypothesis that the CME IIN is similar to randomized networks with the same global properties. The low p-values indicate that the CME IIN is quite distinct from the randomized networks and unique in its local structure.

To further quantify the significance of the local structural elements in the IIN, we evaluate the relative abundance of all 6 different types of 4-node motifs in the network. As shown in Table 2, their abundance in the IIN differs significantly from randomized networks. In particular, 4-node hubs with one shared interface binding to four non-shared interfaces are abundant in the actual IIN, and 4-node chains with four shared interfaces forming a linear chain of interactions are suppressed. The low abundance of these motifs is expected from network specificity optimization [37]. Interestingly, 4-node squares formed by four shared interfaces binding as in A1-B1, B1-A2, A2-B2, B2-A1 are also enriched in the IIN, forming a motif that has high specificity and can arise from gene duplication. Collectively these abundance shifts suggest that the local structure of the IIN is not random but reflects distinct evolutionary mechanisms acting on its topology.

Moving to the global properties of the IIN, we find that the distribution of binding partners per interface follows a power law quite well, with most interfaces having only a single binding partner (Figure 4b). The degree distribution of the IIN is constrained by the parent PPI degree distribution but not fully determined by it, as a PPI can theoretically give rise to many IINs with distinct numbers of nodes and connectivity (but each IIN uniquely defines its parent PPI). Hence a power-law distribution of the number of partners per interface is not a trivial outcome of having a power-law distribution of the number of partners per protein. We do expect that the number of nodes in the IIN will increase relative to the PPI and therefore the number of partners per node will be split between more nodes (assuming the number of edges stays about the same). How exactly the degree distribution changes from PPI to IIN then depends on whether it is mostly the highly connected hub proteins that are split about equally between multiple interfaces, or whether some interfaces retain large portions of binding partners and several single partner interfaces are created. In the CME network, the maximally connected node in the PPI (actin) is split between interfaces, but not evenly, such that one interface retains the majority (16) of the 23 binding partners. Overall, the IIN contains a significant number of highly connected nodes, just as in the PPI. The biggest change in the degree distribution from the PPI to the IIN was the formation of many single-partner interfaces in the IIN, whereas a protein in the PPI was more likely to have at least 3 partners. We discuss further below whether these trends might be conserved in other IINs.

**Figure 5:**
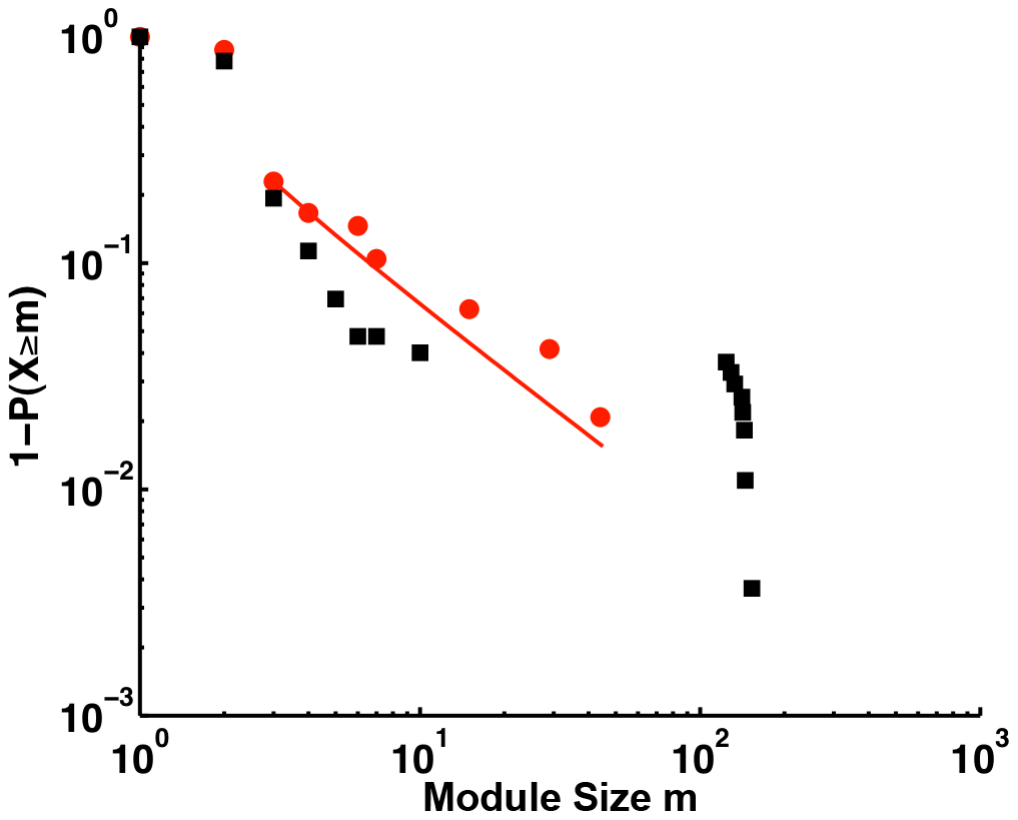
Cumulative distribution of module size *m* in the IIN. The module size is the number of interfaces in a connected fragment of the IIN. The cumulative distribution is shown rather than the probability distribution because of the small sample size. For a power law probability, *p(m)*∼1/*m* ^γ^, the cumulative distribution P*(m)*∼1/*m* ^γ−1^ must also be a power law with an exponent γ−1. The best power-law fit to the probability distribution is for m_min_ = 3 and γ = 1.94, giving a p-value of 0.21 (red line). The black squares are the distribution for a set of networks that have the same size and degree distribution of the CME IIN, but with randomly reconnected edges. The randomized networks separate into one large component and a few small ones, with a gap between ∼10 and 100, in contrast to the modular structure of the CME IIN.

Another distinguishing feature of the IIN is its fragmentation into modules, unlike the densely connected PPI. Compared to randomized networks, the CME IIN has a diverse distribution of module sizes, with many small fragments, whereas randomized networks all have a single giant connected component alongside many small fragments (Figure 5). In fact, the number of interfaces in each CME fragment again appears to follow a power law distribution with an exponent of about −2 (Figure 5). As a result, isolated small modules dominate, but larger connected networks even at the interface level are not uncommon. One must keep in mind, though, that here we focus on only a limited, functionally defined module. In future studies, it will thus be interesting to examine other IINs resolved at the same level of detail.

### IIN modules and interface clustering

The modules in the IIN start to show clustering of interfaces with shared properties, although to varying degrees. In Figures 3a and 3b, we colored the interfaces according to specific domain types that are repeated in the network: PRDs and SH3 domains; EH domains and NPF motifs; phosphorylation sites and kinase domains; clathrin boxes; acidic domains; and subunit-subunit interfaces. As seen in Figure 3a, at the PPI level these interface types are mixed (i.e., distributed across different proteins); by contrast, we find them to be clustered into separate IIN modules. In randomized networks such clustering is not observed. This clustering of interface types reflects the need for binding interfaces to maintain high specificity towards their complementary binding partners and against binding towards unrelated interface sequences [37]. We note that our choice of defining all phosphorylation sites as distinct interfaces places them all in the same module (see Methods), whereas an alternative definition (for example, treating any phosphorylated residues overlapping with other interfaces as forming shared interfaces) would distribute some of them throughout the network. By contrast, the actin ACT1.2 interface is part of a large module with significant heterogeneity in domain types, as discussed further below. Because these binding interfaces do not all contact the same residues of the ACT1.2 interface, they do not all classify according to a single domain type. The convergence of these distinct partners to bind a single protein surface seems more likely a result of functional selection rather than duplication and divergence [38].

## Discussion

### IIN topology

The IIN shares some of the scale-free characteristics of PPI networks [30], yet differs markedly in a number of network topological properties, including a lower average degree of the IIN and a more fragmented structure. While strictly applying only to the CME IIN, we expect many of these results to be conserved in IINs derived from larger PPI networks. First, the comparison of the IIN structure with randomized networks suggests evolutionary pressure acting on the IIN to prevent both giant connected components and a high clustering coefficient (where two interacting interfaces have the same partners). Second, interfaces that have only a single partner should be robustly conserved even for larger networks because they frequently mediate inter-subunit contacts (see light green nodes in Figure 3), and can evolve to high specificity [37]. A noticeable increase in singly connected nodes when transitioning from PPI to IIN would contribute to a steep power law-type degree distribution as a general trend. If a few hub interfaces and many single interfaces were maintained in other IINs, their degree distributions would resemble power laws. The degree distribution of interface partners is noteworthy because power-law distributions indicate networks robust against attacks on specific nodes [39], as would occur from mutations to specific binding surfaces or targeting by binding inhibitors.

In a separate study, we will pursue the hypothesis that the structure of the IIN evolved to minimize nonspecific binding, and that therefore the network features of the IIN encode important physical and biological functions of the proteins. Since minimization of nonspecific binding is a physical pressure common to all proteins [37], [40], we would predict that these topological features would then be conserved in all IINs, not just for CME proteins.

### CME Biology

A number of distinct patterns emerge in the CME IIN. From the degree distribution of the IIN, we can contrast the properties of single interfaces from hub interfaces. More than a quarter of the single partner interfaces come from interfaces between subunits of a multi-subunit complex like ARP2/3 [41]. Dimerization interfaces also tend to be single partner interfaces. The most highly connected interfaces, or hub interfaces, are a surface on the actin protein with 16 partners, and several SH3 domains. The actin surface is distinct from the SH3 domains in that its binding partners do not all conform to the same binding type. The binding interface ACT1.2 is a relatively large and flat region spanning parts of subunits I, II and III of actin (Figure 1a), where not all binding partners use the same set of residues to stabilize their interactions, but the overlap is still significant. While it is certainly possible that with additional residue information this interface could be refined and split into more than one binding site, the extensive sharing of the ACT1.2 interface is consistent with earlier studies that found flat interfaces to provide a better platform for binding a large variety of partners [42], as geometrical packing need not be as optimized. Furthermore, we note that the nucleotide binding state of actin strongly tunes the affinity for its distinct partners.

The IIN overlaid on the PPI reinforces that many of these endocytic proteins are able to bind multiple partners simultaneously because of the number of distinct interfaces. This directly observable insight would be lost if one only categorized protein-protein interactions (i.e., edges in the PPI) as either competing or not, since many proteins have multiple shared interfaces. The interface assignments also highlight redundancy in the network, where the recruitment of a particular protein during the endocytic pathway could happen via multiple mechanisms, as many of the proteins are chimeras of the most frequently represented domains [43] in this network. Of the endocytic proteins, 16% contain SH3 domains, whereas in the entire yeast proteome <1% of proteins contain SH3 domains.

The designation of distinct domains on each protein allows one to contrast the specific structural elements present in these CME proteins versus CME proteins in other organisms. Much of the CME pathway between yeast and mammals is conserved. However, a major distinction is that CME in yeast requires the actin network to initiate the membrane invagination [44], whereas in mammals the actin network is engaged only in some cases in the later stages of vesicle budding [45]. It is interesting to note that of the 9 CME proteins in yeast without functional homologs in mammals [26], all but one (PAL1) engage in SH3 or PRD interactions (LSB3, LSB4, LSB5, BBC1, AIM21, BSP1, AIM3, APP1). This finding is statistically significant, having only a ∼0.15% probability to occur by chance (as determined by the probability of choosing at random 8 or more proteins out of 9 that have SH3 or PRD interactions, with 22 candidates among the 56 proteins of the CME network). The SH3 domains in the CME network recruit proteins throughout the progression of the vesicle budding process after the initial clathrin coat assembly [35]. The abundance of SH3 domains in yeast CME proteins likely reflects the central role of their interactions in connecting the growing clathrin coated pit to the actin cytoskeletal network of yeast.

Distinguishing interface domains in each protein also enables direct visual identification of multi-interface proteins that act to bring together multiple proteins with different functions, which again is not possible if one only marks edges in the PPI as competing. Both PAN1 and SLA1 have many interfaces that can connect simultaneously to both the scaffolding proteins of the clathrin pit formation (through PAN1's EH domain [32] and SLA1's clathrin box [46]), and to the actin polymerization proteins via PRDs, SH3 domains, and acidic domains. LAS17, on the other hand, does not connect directly to the scaffold proteins of clathrin pit formation but rather has distinct interfaces to bind both SH3 proteins and the ARP2/3 complex. While the role of LAS17 is not fully understood [26] and appears to involve both activation and inhibition of actin branching [47], the designated interface-interface pairs provide a basis for grouping the many functions of this protein along with distinct CME proteins according to domain types (including PRDs, acidic domains, the C-helix and WH2 domains). Lastly, some multi-interface proteins in the network, such as Arc15 and Arc19, contain only subunit-subunit interface domains, indicating that they function as structural components of a multi-subunit complex.

### Designing selective drug targets and preventing cross-reactivity

Designing any ligand, and in particular a drug molecule, to bind exclusively to its intended target without cross-reactivity requires not only positive selection for the specific target but also negative selection against related targets [37]. The clustering of interfaces in modules in the IIN provides a tool for predicting which binding partners of an interface are the most selective for its surface and do not bind to related domains. For example, both RVS167.2 and the SH3 domain of YSC84/LSB4 (YSC84.1) bind several of the same PRDs. Obtaining target specificity for only one of those interface sites benefits from knowing which PRDs are specific to only one of these interfaces. The interfaces VRP1.0, BSP1.3, and ABP1.1 that bind RVS167 but not YSC84, and ABP1.5, LAS17.6 and AIM21.0 that bind YSC84 but not RVS167, could be used as templates for targeting only one of the two SH3 domains.

### Predicting response to mutations and pathway inhibition/activation

Collectively, the information on interface connectivity and protein connectivity combined in a network format provides important guidance for the selective inhibition or activation of specific pathways, for drug targeting, and for predicting response to surface mutations. The PPI network is essential for identifying which proteins interact in a functional pathway, but the details of the IIN allow one to isolate specific binding sites while conserving the functionality of other sites. The IIN also allows one to predict how drugs designed as roadblocks along a certain pathway could be bypassed by alternate available interface interactions.

For example, one might expect that inhibiting or mutating ‘hub’ interfaces, much like knocking out ‘hub’ proteins, would induce a more severe phenotype. RVS167.2 is found to interact with 12 PRD interfaces as part of the PRD-SH3 IIN sub-network in the top right of Figure 3b. These interactions are not immediately apparent in the PPI network, lacking interface resolution. While early studies [34], [48], [49] already pointed to the prevalence of these interactions, their functional importance and temporal recruitment in endocytosis is emerging only now [35], [50], [51]. Mutations of the SH3 domain of RVS167 that leave its membrane shaping BAR domain intact still significantly alter the endocytic phenotype [50]. A substantial phenotypic response to such a localized mutation would be anticipated from the IIN because removing that particular node removes multiple edges. However, the fragmented and clustered structure of the IIN also provides a more detailed perspective on the response to deleting this node. Although targeting the SH3 domain of RVS167 would inhibit 12 RVS167 binding interactions, one can see in the IIN that most of those interface partners can also bind to alternate SH3 domain containing proteins and all of the interface partners are on proteins with a PRD that can bind an alternate SH3 domain. These alternate pathways may help explain why mutations of the SH3 domain of RVS167 do not eliminate endocytic function in yeast [50].

### Missing components from predicted mutational responses

The inhibition of particular binding sites would have unexpected results if there were nodes or edges missing from the network. For example, truncation of the clathrin N-terminal domain (CHC1.1 in Figure 3b) was accurately predicted to cause a severe endocytic phenotype by preventing recruitment of clathrin to the membrane. However, when the known binding sites on the N-terminal domain were mutated, the expected result was not observed, and this led to the identification of duplicate binding sites in the N-terminal domain [52]. We do note that the CME IIN overlaid on the PPI proposes another mechanism for recruitment of clathrin to the membrane via binding of the clathrin light chain (CLC1) to SLA2, which can then bind the membrane or other membrane bound proteins. This interaction may be too weak to recruit clathrin on its own, or SLA2 may be too small to bridge the large separation from the clathrin light chain to the membrane.

### Cooperative binding and opposing mutations

The IIN also indicates sets of opposing or reciprocal mutations, or truncations that should result in the same phenotypic response. The prediction of the identical responses assumes that the binding partners of the targeted interface act independently of other binding partners. The extent to which these assumptions are violated could suggest allostery or cooperativity between the affected partners. Identifying interfaces for reciprocal mutations could then offer a tool for testing cooperativity or dependence between binding interactions or for identifying missing interfaces. In the clathrin CHC1.1 interface example given above, the IIN would predict a reciprocal mutation to all five clathrin boxes to give the same phenotype as the removal of the CHC1.1 interface. If, instead, clathrin were still recruited to the membrane, then one expects other clathrin N-terminal binding sites to be missing from the network.

In another module, the EH domains are shown with their NPF motif binding partners. Based on the specificity of these interfaces for one another only in the IIN, one would expect that mutations to either the NPF motifs (including all copies) or to the EH domains (including all copies) would generate the same phenotype. The extent to which they do not match would first indicate possible missing nodes from the network. Alternatively, the result could indicate that one of these domains acts cooperatively with another domain to affect the global behavior of the protein, not just this specific interaction.

In terms of the anticipated biological response to mutation of either the EH domain or the NPF motifs (assuming independence), this interaction helps stabilize a scaffold of proteins at the membrane that recruits the clathrin trimer. From the IIN combined with the PPI, cutting these edges out of the network would not prevent any of the proteins from connecting to the membrane or the early coat module, as PAN1 could still connect via SLA2 and EDE1 via SYP1. Clathrin and actin would still be recruited normally. What this mutation should affect is crosslinking between these proteins and therefore clustering of these proteins in one place on the membrane. If crosslinking and clustering of proteins is necessary for efficient coat formation then eliminating these interactions could decrease or slow down clathrin-pit formation.

### Challenges in IIN construction

As one of the main challenges in IIN construction, there is more than one way to define whether a binding interface on a protein is shared between multiple binding partners or is completely distinct. The two main criteria we use to characterize shared and distinct interfaces are (1) if the same residues are present in both interfaces, (2) if the binding of one protein partner would interfere with the binding of another partner due to structural overlap or allosteric effects. Both criteria are important to the function of the proteins in the cell. Concerning the first criterion, the sequence makeup of the interface is central to achieving binding specificity, as even proteins with the same domain structures do not necessarily share the same partners [35]. Furthermore, the residues involved in a binding interaction are not only important for binding to their specific partner but also for avoiding the formation of nonfunctional interactions with the other proteins in the cell [37]. This negative selection on an interface can contribute to optimizing the specificity and strength of functional binding interactions [25]. Concerning the second criterion, determining whether two potential binding partners can both bind at the same time to form a trimer is important for modeling the dynamics of protein association, as competition for binding partners will affect concentrations of available protein. The same is true if a protein has repeated copies of the same domain and can therefore bind multiple copies of the same binding partner. However, it may not always be possible to assign distinct interfaces that meet both criteria of sequence specificity without any steric obstruction and therefore in this work we consistently emphasize residue detail where the information is available. Otherwise we did use competition for binding partners as grounds for defining shared versus distinct interfaces. In future work it would be valuable to annotate both the residues involved in each interface as well as whether each pair of distinct interfaces on a protein can bind their partners simultaneously.

### Lessons for high-throughput interface assignment

The procedure of manually assigning interfaces has also highlighted some important issues for consideration in computerized interface assignment. For one, residue overlap does not necessarily mean that proteins compete for binding to the protein, as demonstrated by multi-subunit complex formation (Table 3). None of the interface interactions within the complex would be considered shared because they are all bound together at the same time. The majority of interfaces do not overlap, but ∼30% of the bound partners share one or more residues in their interactions. Most commonly the overlap was only one or two residues, and the corresponding percentage of the interface varied substantially depending on the size of the protein. Thus it seems reasonable to allow 1–2 residues of overlap before defining interfaces as shared. This policy is also consistent with the assignment of different domains as distinct interfaces, even though the 3D structure of the protein might produce some residue overlap between two distinct domains. We note that here we did not use a strict cutoff in our assignments because through manual curation we treated each interaction on a case-by-case basis, merging residue level detail with experimental data on simultaneous binding partners.

**Table 3:** Multi-protein complexes and interface residue overlap.

	0 residues overlapping	1 residue overlapping	>1 residue overlapping
**Proteasome subunits (1RYP.pdb) 4A cutoff**	62%	21%	17%
**Proteasome subunits 3.5A cutoff**	82%	12%	6%
**Arp2/3 subunits (1K8K.pdb)4A cutoff**	54%	17%	29%
**Arp2/3 subunits 3.5A cutoff**	68%	16%	16%

The subunits of multi-protein complexes bind together simultaneously and therefore these subunit proteins are not competing to bind to the same interface. For each subunit *S* in the complex, we test all pairs of its binding partners for sharing binding residues on the surface of *S*. Each binding pair then has *n* = 0, 1, 2 etc. overlapping residues. Whereas most binding pairs do not share interface residues, clearly there is some overlap. If one accounts for specific atoms in an interface rather than residues, the overlap decreases but is still present. For the proteasome, there are still 22% and 7% of interface pairs that share atoms (at 4 Å and 3.5 Å cutoffs, respectively).

For attempts at homology modeling or docking, it would first be useful to assess how reliable a purported interaction might be. Particularly in the case of interactions involving subunits of multi-protein complexes, many of the interactions are actually indirect. Arp2, for instance, has relatively high homology to actin and shares several binding partners; however, Arp2 acts as part of a multi-subunit complex and binds to these shared partners (such as LAS17) in distinct ways. Also, higher thresholds for sequence similarity could be warranted in particular cases, such as SH3 domains, where small variations in sequence distinguish specific from nonspecific partners [25].

One of the major distinctions between the procedure used here and current automated methods is the inclusion of detailed information on binding interfaces between proteins from biochemical studies, not just from high-resolution protein structures. This information preempts or complements the use of homology or docking models of protein interactions. Unfortunately, the domain or interface details from these studies is not collected in a convenient database, whether it is the specific residues that comprise the interface or more general information on inhibition or competition between binding partners. There are also ambiguities and inconsistencies in existing data that are difficult to resolve without combining multiple literature resources in a coherent analysis. Nevertheless, mining these data would provide a valuable resource for generating more complete networks of interface-interface interactions.

## Methods

### Defining the PPI

Our protein list is composed of 56 proteins that were selected because they all participate in the yeast clathrin-mediated endocytosis pathway and have been identified as central components [26]. We downloaded the physical interaction partners of the 56 proteins of the endocytic functional module in yeast via the Saccharomyces Genome Database (SGD) [29] interaction list compiled from BioGRID [28] and directly from the IntAct, MINT, DIP, and BIND protein interaction databases. We kept only the interactions between the subset of 56 proteins to define the initial set of experimentally determined protein-protein interactions. We disregarded genetic interactions, as they do not imply that the proteins directly interact with one another, but rather that their expression or phenotype is correlated. The overlap in databases was quite large for these proteins, with BioGRID containing the largest number of interactions and missing interactions coming not from missed references but from missed interactions within the same references.

### Data collection procedure

Given a PPI network, the first step in assigning the binding interfaces was collecting information on the particular proteins from the SGD [29]. The SGD combines information from various databases on each yeast gene. The major data sources we used were the list of referenced physical interactions loaded from the various PPI databases and the available PDB structures. The protein tab also provides a useful guide to the size, sequence, domain structure, and function of each protein.

### Data collection: Crystal structures

Crystal structures of complexes were available for a few of the protein interactions, including the ARP2/3 complex and several actin binding interactions (shown as black edges in Figure 3). We ensured that we matched the numbered PDB residues (which sometimes started at zero arbitrarily) to the correct sequence region on the protein of interest. For protein homologs from species other than yeast, the sequence alignment is also provided for positioning the interface on the yeast protein of interest. Compared across species, actin has high (87%) sequence homology and structures from other species were simply used as proxies for the expected interaction in yeast. To assign residues involved in the protein interfaces from a PDB complex we used a 4-Å cutoff between non-hydrogen atoms and required that at least 3 residues contacted one another in each interface. Cofactors such as metal ions and water molecules were not considered in assigning whether two proteins interacted or which residues formed the interfaces. Some of the protein structures had multiple missing residues for crystallization purposes, such that the assigned interface may be smaller than in the complete protein. By using the PDB structures we eliminate all indirect interactions that are often assigned to protein subunits of a large complex in high-throughput AP/MS and PCA. We did not use any predicted models of protein complexes [23] because direct information was generally available through literature studies and because protein homologs (e.g., Arp2 and actin) do not always share the same set of binding interactions.

### Data collection: Biochemical data

In most cases, crystal structures were not available and instead the literature references from the PPI databases were used to assign interfaces. Binding to proteins outside the endocytic network, as listed in the SGD, was ignored. Nearly all of the edges to which we assigned interfaces were implicated as binding in more than one experiment. We have collected all the justifications for each assignment into a spreadsheet with references (see Table S1), categorized the support for each interface assignment with edge colors in Figure 3, and below we describe additional criteria we used to define the interfaces for the specific cases of kinase binding and SH3 domains binding to PRDs.

### Data collection: SH3-PRD interactions

Several of the endocytic proteins have SH3 domains (BZZ1, ABP1, LSB3, LSB4, RVS167, BBC1, MYO3, MYO5, and SLA1) and PRDs to which SH3 domains bind (VRP1, LAS17, MYO5, APP1, AIM21, AIM3, SCD5, BBC1, ABP1, ARK1, PRK1, INP52, SCP1, BSP1, SLA1, SYP1, GTS1). We took advantage of several large-scale studies [34], [35], [48] focused on identifying which PRDs bind to which SH3 domains by compiling all interactions noted for our 56 proteins (including those interactions missing from the PPI databases). Tonikian et al. [35] provide the most recent and comprehensive study to identify PRDs by combining data from three independent experiments. We assigned the PRD and SH3 interfaces if the interactions were observed by Tonikian et al. and at least one other experimental study. As one exception to this criterion, if there is only one supporting experiment, yet that experiment found a different PRD site, then the interface was left unassigned. Lastly, if more than two references reported binding and the PRDs were different, the two PRDs were combined into one binding site. Binding multiple PRDs on the same protein has been experimentally demonstrated [34], but Tonikian et al. only report the most likely PRD, so this does not rule out additional PRDs. We merged the two SH3 domains of BZZ1 to improve the consensus of their binding partner interfaces but kept the two SH3 domains of SLA1 separate. We separated the multiple PRDs of LAS17 into distinct binding sites as multiple lines of evidence implicated specific binding partners for specific regions. These details are collected in Table S1, under tabs 2 and 3.

### Data collection: Phosphorylation

The endocytic protein subset contains three kinases (ARK1, PRK1, AKL1) and similar to the SH3 domains, the specificity of kinases for their phosphorylation targets has also been studied at large scale [53], [54]. We here again compiled the interactions from Breitkreutz [53], Mok [55], and Ptacek [54] and their collaborators (again including some interactions missing from the PPI databases), and assigned the interactions if the binding was reported in at least two references. Because most of the targets in Mok et. al. [55] were predicted but not verified, we included these sites as references only if they were also experimentally tested or observed in previous mass spectrometry experiments. These details are collected in Table S1, under tab 4.

**Table 4:** File storage of interface overlap for the subunit ARC40.

	ARC19	ARC15	ACT1	LAS17	MYO5	MYO3	PAN1	CRN1
**ARC19**	_	0	0	0	0	0	0	0
**ARC15**	0	_	0	0	0	0	0	0
**ACT1**	0	0	_	1	1	1	1	1
**LAS17**	0	0	1	_	1	1	1	1
**MYO5**	0	0	1	1	_	1	1	1
**MYO3**	0	0	1	1	1	_	1	1
**PAN1**	0	0	1	1	1	1	-	1
**CRN1**	0	0	1	1	1	1	1	-

In this 8×8 matrix, each row/column represent an interface for binding of ARC40 to each of its 8 partners. Overlapping and non-overlapping interfaces are indicated by 1 and 0, respectively. Diagonal entries are ignored. In the case of self-binding the interaction could be mediated by the same surface on both protein copies (1 matrix entry) or by two distinct surfaces (2 entries). The shared surface of ARC40 is the region that binds to the acidic domains of LAS17, MYO5, MYO3, PAN1, and CRN1. This surface is not specifically known, but the homology of the partners suggests they all bind to the same place. Actin is included as binding to this shared surface because its binding is impeded by binding of LAS17 to ARC40 and there is no additional information on the residues involved in the interactions to determine if the interference is direct or allosteric.

### Data collection: Unassigned and removed edges

In some cases the data implicating two proteins as interacting only came from high-throughput studies and these interactions were generally unassigned. Others came from a literature source that did not isolate binding interfaces, with no additional evidence available from homologs or functionally related proteins. Edges that were identified between the ARP2/3 complex subunits and other proteins were considered indirect if PDB structures or biochemical evidence implicated a specific subunit in the direct interaction. For a few interactions, evidence from the literature suggested that such proteins did not bind directly to one another upon further investigation, and as a result these edges were removed. We note these in the interaction table. For example, we were unable to find any evidence for the protein RVS161 forming direct physical interactions with any proteins other than RVS167. Furthermore, there was some biochemical evidence suggesting that proposed edge interactions were mediated via RVS167 rather than directly through RVS161 [56], as they operate as an obligate dimer.

### Data collection: Adding edges

We added 9 new edges to the network to account for literature studies providing evidence for the binding interactions. These were largely actin related interactions that lacked references in the PPI databases but have been well established as functionally important binding partners of actin. One was an SH3-PRD interactions defined in two separate publications that were missing from the database.

### Data collection: Membrane binding activity

Several of these proteins have domains known to bind at the membrane that are important to their function in endocytosis. Therefore we pointed these out on the protein-interface interaction network in Figure 3a to facilitate the prediction of functional responses to mutation.

### Distinguishing unique interfaces: Residue level description

As the first criterion to assign an interface, we used the residues involved in the binding, if available. Specific residues were available from PDB structures and for several peptide motifs like PRDs [34], [35] and clathrin boxes [32], [46], [57]. If two partners of a protein bind to an interface using some overlapping residues we did not automatically classify the interface as shared. There are two reasons for this decision, the main one being that sharing one or a few residues does not mean those two proteins cannot bind simultaneously. To demonstrate this point we calculated the percent of distinct interface pairs within a multi-subunit protein complex that had overlapping residues. For each of the complexes we considered, there are some pairs of interfaces that have one or more residues in common (Table 3). Even if the interfaces are defined at the atomic rather than residue level, there is still a fraction of atoms within the cutoff distance of both binding partners. The second main reason is that even if the binding partners cannot bind simultaneously, the specificity and stability of their interactions may be mediated through chemically distinct binding sequences. For example, we chose to treat a kinase's phosphorylation binding site as distinct from other protein binders that may interact with the phosphorylated residue because of their distinct binding modes. However, if the residue overlap is substantial, as is the case for many proteins that bind to actin in similar but not identical ways, then the interface is considered shared.

### Distinguishing unique interfaces: Domain level description

When the specific residues of the folded protein interfaces were not available, the next description of the interface was the domain structure represented by sequential sequence residues (e.g., the SH3 domain contained in residues 1–51). These domains were generally identified in biochemical studies and the sizes of the domains varied from a few residues (e.g., clathrin boxes) to hundreds of residues (e.g., coiled-coil domains). In some cases the assigned interfaces may not represent a known domain but they are designated as unique interfaces because they do not overlap with any of the protein's other binding partners. Lastly, if residue level detail is not available, then the fact that two binding partners are competing with one another is used as justification for listing the interface as shared.

### Shared versus distinct interface summary

To summarize, we did not use a strict cutoff of overlapping residue numbers for defining shared versus distinct interfaces. All subunits of a multi-subunit complex were assigned distinct interfaces for these inter-subunit contacts because they could clearly bind simultaneously. This is despite the fact that pairs of proteins could have as many as 10 overlapping residues if a long disordered region of a protein sat at the seam of an interaction between two other proteins. For most biochemical studies, stretches of residues were identified and shared interfaces were assigned when proteins bound to overlapping stretches of residues and there was no evidence that they could bind simultaneously. For the distinct surfaces in actin, there was in some cases overlap between residues, but there was also evidence that the proteins could bind simultaneously. For example, several actin binding proteins bind to the actin filaments, and therefore they can bind simultaneously with the actin-actin binding interactions, despite overlapping with some residues.

### Matrix representation of interface interactions

In representations simpler than the IIN, edges in the PPI network have been marked as shared. To extend the representation to full interface assignments, one must keep track of possible overlap in all pairs of binding interactions for each protein. Given a protein that has *k* binding partners, there are *k*(*k*−1)/2 possible pairs of partners sharing an interface. To keep track of the interface assignments, each protein had its own file with a *k*-by-*k* matrix indicating the overlap between the *k* binding partners (Table 4). The diagonal entries are null and the off-diagonal entries of the symmetric matrix are 0 if the two partners use separate interfaces and 1 if the two partners use the same interface. Some protein-protein interactions are controlled by more than one set of interfaces and would require an additional entry into the matrix. The binding interfaces from each protein can then be consolidated into a network representing a connected set of interface interactions. We note that in a matrix representation it is possible to define a case where one interface overlaps with two others that do not overlap with each other, and this detail cannot be captured in a simple interface network picture. This would be the case, e.g., if two proteins A and B bind to two distinct parts of a protein X and the third protein C binds across those two complete interfaces on protein X. However, this issue can easily be fixed by splitting protein C's interface into two interfaces to bind the two parts of protein X. For example, this splitting was done for LAS17's CA region that binds to ARP3 through both its C interface and its separate A interface [47].

### Network properties

We evaluated clustering coefficients of our networks using the expression [58]

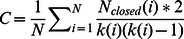
where *N_closed_*(*i*) counts how many distinct pairs of the *k*(*i*) partners of interface *i* have an edge between them to form closed triangles with node *i*. Self-loops were ignored in this calculation. We also use a global clustering coefficient *C_global_* as the number of distinct closed triangles *N_triangle_* in the network divided by the total number of distinct triplets,
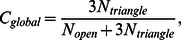
with *N_open_* the number of open triplets.

We computed degree distributions, *p(k)*, where *k* counts the number of partners per node (either protein or interface), and *p(k)* is the probability for finding a node in the network with that degree. For the degree distribution, we note that we treated self-loops as a single partner, rather than the standard method of treating a self-loop as counting as degree of 2, so that the degree would reflect the physical number of binding partners per protein (or interface).

### Network motifs

We enumerated the 4-node motifs present in our networks by identifying all distinct sets of 4-node subgraphs that are connected by at least one path (each node in the subgraph can be reached by the others). There are six distinct 4-node subgraph architectures [59] and we note that they are all counted mutually exclusive to one another, i.e., a set of 4 nodes uniquely classifies as one of the six subgraphs. A single node may belong to more than one 4-node subgraph. Hub and chain motifs have 4 nodes connected by 3 edges, flag and square motifs have 4 nodes connected by 4 edges, and the other two 4-node subgraphs contain 5 and 6 edges.

### Randomized networks

To generate networks that shared the same number of interfaces, edges, and the same degree distribution as the IIN in Figure 3b, we used the Monte Carlo method of Maslov and Sneppen [60]. Specifically, in a trial move two interfaces were selected randomly and a partner from each of these interfaces was randomly selected. The partners were then swapped between interfaces, unless one of these new edges already existed, in which case the move was rejected.

### Power law density fitting

We fit our degree distributions to power laws using the maximum likelihood method, where the discrete data is fit to a power law distribution x^−γ^/ζ(γ) normalized over the range x≥x_min_ [31]. We measure the goodness-of-fit using the Kolmogorov-Smirnov metric and calculate the p-value for the data being drawn from a power law density using the method of ref. [31]. For the p-value calculation, our null hypothesis is that the data is drawn from a power-law density. Therefore, a small p-value of <0.05 would reject this null hypothesis and demonstrate that our data is not described by a power law. A large p-value, on the other-hand, indicates that the data is consistent with the hypothesis that it was drawn from a power law distribution.

## Supporting Information

Table S1 Five sub-tables list (1) the justifications and literature sources used for the interface assignments, including network edges left unassigned, and details about the (2) SH3 domain, (3) PRD (4) kinase interactions and (5) membrane binding domains.
(XLSX)
